# Supplementary material for: Negative regulation of conserved RSL class I bHLH transcription factors evolved independently among land plants
Source: eLife. 2018 Aug 23;7:e38529. doi: 10.7554/eLife.38529 (PMC6141232; doi:10.7554/eLife.38529)
Supplement: Supplementary file 3. [file elife-38529-supp3.rtf]

Supplemental file 3: 5'RLM RACE PCR sequence data

The 5' RLM RACE PCR products were cloned into pGem-T vector prior to sequencing. The pGemT vector sequence is indicated in red, RNA primer sequence in blue and sequence corresponding to the MpRSL1 transcript cleavage product in black.

>colony 1
NNNNNNNNNNNNANGGCNATTGGGNNNACGTCGCATGCTCCCGGCCGCCATGGCGGCCGCGGGAATTCGATTGGACACTG
ACATGGACTGAAGGAGTAGAAATTCTCTTACATCCACCTGTTCTAAGACTGTAGACGCCATTAACCCTGGGCCTCTACCT
TACACCAAGTCGAATTCACGACATGCCGTCGGGCCGGCTTTGAACACCAATCTGATGCCGCGAGCCCGGCAGGGCAGTGC
GAACGATCCTCAGAGCATAGCAGCTCGCCATCGGAGGGAACGCATCGGCGAGCGACTGAAGACTCTACAAGATCTTGTTC
CTAATGGTTCCAAGGTAGACTTGGTCACCATGCTTGAAAAGGCAATCACTAGTGAATTCGCGGCCGCCTGCAGGTCGACC
ATATGGGAGAGCTCCCAACGCGTTGGATGCATAGCTTGAGTATTCTATAGTGTCACCTAAATAGCTTGGCGTAATCATGG
TCATAGCTGTTTCCTGTGTGAAATTGTTATCCGCTCACAATTCCACACAACATACGAGCCGGAAGCATAAAGTGTAAAGC
CTGGGGTGCCTAATGAGTGAGCTAACTCACATTAATTGCGTTGCGCTCACTGCCCGCTTTCCAGTCGGGAAACCTGTCGT
GCCAGCTGCATTAATGAATCGGCCAACGCGCGGGGAGAGGCGGTTTGCGTATTGGGCGCTCTTCCGCTTCCTCGCTCACT
GACTCGCTGCGCTCGGTCGTTCGGCTGCGGCGAGCGGTATCAGCTCACTCAAAGGCGGTAATACGGTTATCCACAGAATC
AGGGGATAACGCAGGAAAGAACATGTGAGCAAAAGGCCAGCAAAAGGCCAGGAACCGTAAAAAGGCCGCGTTGCTGGCGT
TTTTCCATAGGCTCCGCCCCCCTGACGAGCATCACAAAAATCGACGCTCAAGTCAGAGGTGGCGAAACCCGACAGGACNA
TAAGANNNNNNGGCGTTTCCCCCTGGNNNCTCCTTCGTGNNNNTCTCCTGTTCCGACCNGNCGCTNACCGGANNNNNTNN
CCNNCNTTTNTTCCNTTNGNNAGCNNNGNNNCTTTCTCNNANCTNACNCTGTANGNNNNNNTCANNNNNGGNGNAGNNGN
TNCGCNNNCNNNNNGGNCNNNNNNCNNCNAANCNCNNNTNNNNNNNNANNNNNNNNNNNNNNNNNNNANNNNNCNNNGAN
NNNCNANCNNGGNNNNNNNNNNNN

>colony 2
NNNNNNNNNNNNNNNNGGNNNTTGGGNCNACGTCGCATGCTCCCGGCCGCCATGGCGGCCGCGGGAATTCGATTGGACAC
TGACATGGACTGAAGGAGTAGAAATTCTCTCACATCCACCTGTTCTAAGACTGTAGACGCCATTAACCCTGGGCCTCTAC
CTTACACCAAGTCGAATTCACGACATGCCGTCGGGCCGGCTTTGAACACCAATCTGAAGCCGCGAGCCCGGCAGGGCAGT
GCGAACGATCCTCAGAGCATAGCAGCTCGCCATCGGAGGGAACGCATCAGCGAGCGACTGAAGACTCTACAAGATCTTGT
TCCTAATGGTTCCAAGGTAGACTTGGTCACCATGCTTGAAAAGGCAATCACTAGTGAATTCGCGGCCGCCTGCAGGTCGA
CCATATGGGAGAGCTCCCAACGCGTTGGATGCATAGCTTGAGTATTCTATAGTGTCACCTAAATAGCTTGGCGTAATCAT
GGTCATAGCTGTTTCCTGTGTGAAATTGTTATCCGCTCACAATTCCACACAACATACGAGCCGGAAGCATAAAGTGTAAA
GCCTGGGGTGCCTAATGAGTGAGCTAACTCACATTAATTGCGTTGCGCTCACTGCCCGCTTTCCAGTCGGGAAACCTGTC
GTGCCAGCTGCATTAATGAATCGGCCAACGCGCGGGGAGAGGCGGTTTGCGTATTGGGCGCTCTTCCGCTTCCTCGCTCA
CTGACTCGCTGCGCTCGGTCGTTCGGCTGCGGCGAGCGGTATCAGCTCACTCAAAGGCGGTAATACGGTTATCCACAGAA
TCAGGGGATAACGCANGAAAGAACATGTGAGCAAAAGGCCAGCAAAAGGCCAGGAACCGTAAAAAGGCCGCGTTGCTGGC
GTTTTTCCATAGGCTCCGCCCCCCTGACGAGCATCACAAAAATCGACGCTCAAGTCAGANGTGGCGAAANCCGACAGGAC
TATAAAGATACCNAGGCGTTTCCCCCTGGNNGCTCCCNCNTGCGCTCNNNNNNNCGACCCNGCCGCTTANCNGATACCTG
TCNNCNTTCTNCCNNNGGNAGCGNNNNCNTTNCTCANAGCTCACGCNNNANGNANNTCANNNNNNNNNNNNNCNNTTCGN
NNNNNNNTNGNNNNNNNNNANNAANNCCNNNNANNNGNNCNCNNNNNCTNNTCNGNNAACNNNTCNNNNNNN

>colony 3
NNNNNNNNNNNGGGNNNNNGGGNNNACGTCGCATGCTCCCGGCCGCCATGGCGGCCGCGGGAATTCGATTGGACACTGAC
ATGGACTGAAGGAGTAGAAATTCTCTCACATCCACCTGTTCTAAGACTGTAGACGCCATTAACCCTGGGCCTCTACCTTA
CACCAAGTCGGATTCACGACATGCCGTCGGGCCGGCTTTGAACACCAATCTGAAGCCGCGAGCCCGGCAGGGCAGTGCGA
ACGATCCTCAGAGCATAGCAGCTCGCCATCGGAGGGAACGCGTCAGCGAGCGACTGAAGACTCTACAAGATCTTGTTCCT
AATGGTTCCAAGGTAGACTTGGTCACCATGCTTGAAAAGGCAATCACTAGTGAATTCGCGGCCGCCTGCAGGTCGACCAT
ATGGGAGAGCTCCCAACGCGTTGGATGCATAGCTTGAGTATTCTATAGTGTCACCTAAATAGCTTGGCGTAATCATGGTC
ATAGCTGTTTCCTGTGTGAAATTGTTATCCGCTCACAATTCCACACAACATACGAGCCGGAAGCATAAAGTGTAAAGCCT
GGGGTGCCTAATGAGTGAGCTAACTCACATTAATTGCGTTGCGCTCACTGCCCGCTTTCCAGTCGGGAAACCTGTCGTGC
CAGCTGCATTAATGAATCGGCCAACGCGCGGGGAGAGGCGGTTTGCGTATTGGGCGCTCTTCCGCTTCCTCGCTCACTGA
CTCGCTGCGCTCGGTCGTTCGGCTGCGGCGAGCGGTATCAGCTCACTCAAAGGCGGTAATACGGTTATCCACAGAATCAG
GGGATAACGCANGAAAGAACATGTGAGCAAAAGGCCAGCAAAAGGCCAGGAACCGTAAAAAGGCCGCGTTGCTGGCGTTT
TTCCATAGGCTCCGCCCCCCTGACGAGCATCACAAAAATCGACGCTCAAGTCAGAGGTGGCGAAACCCGACAGGACTATA
AAGATACCANGCGTTTCCCCCNNNNAGCTCCCTCGTGCGCTCTNCTGTNCNANCCTGNCGCTTANCNGNATANCNGTCCN
NNTTCTCCCTTNNNGNNCGNNNNNTTTCTCNNAGCTCANGCNGTAGNNNNTCANTNNNNNNNNNNNNNNCNNNNNNNCTG
GNNTGNNNNNNCNNNNCCCNNNNANNNNCNCNNNNNNNNNNNNNANNNNCNNNNGANNNCANNCN

>colony 4
NNNNNNNNNNNNTANGGCGAANTGGGNNNACGTCGCATGCTCCCGGCCGCCATGGCGGCCGCGGGAATTCGATTGGACAC
TGACATGGACTGAAGGAGTAGAAATTCTCTCACATCCACCTGTTCTAAGACTGTAGACGCCATTAACCCTGGGCCTCTAC
CTTACACCAAGTCGAATTCACGACATGCCGTCGGGCCGGCTTTGAACACCAATCTGAAGCCGCGAGCCCGGCAGGGCAGT
GCGAACGATCCTCAGAGCATAGCAGCTCGCCATCGGAGGGAACGCATCAGCGAGCGACTGAAGACTCTACAAGATCTTGT
TCCTAATGGTTCCAAGGTAGACTTGGTCACCATGCTTGAAAAGGCAATCACTAGTGAATTCGCGGCCGCCTGCAGGTCGA
CCATATGGGAGAGCTCCCAACGCGTTGGATGCATAGCTTGAGTATTCTATAGTGTCACCTAAATAGCTTGGCGTAATCAT
GGTCATAGCTGTTTCCTGTGTGAAATTGTTATCCGCTCACAATTCCACACAACATACGAGCCGGAAGCATAAAGTGTAAA
GCCTGGGGTGCCTAATGAGTGAGCTAACTCACATTAATTGCGTTGCGCTCACTGCCCGCTTTCCAGTCGGGAAACCTGTC
GTGCCAGCTGCATTAATGAATCGGCCAACGCGCGGGGAGAGGCGGTTTGCGTATTGGGCGCTCTTCCGCTTCCTCGCTCA
CTGACTCGCTGCGCTCGGTCGTTCGGCTGCGGCGAGCGGTATCAGCTCACTCAAAGGCGGTAATACGGTTATCCACAGAA
TCAGGGGATAACGCANGAAAGAACATGTGAGCAAAAGGCCAGCAAAAGGCCAGGAACCGTAAAAAGGCCGCGTTGCTGGC
GTTTTTCCATAGGCTCCGCCCCCCTGACGAGCATCACAAAAATCGACGCTCAAGTCANAGGTGGCGAAACCCGACAGGAC
TATAANATACCAGGCGTTTCCCCCTGGNAGCTCCNCGTGCGCTCTCCTNNNNGACCCTGCCGCTTANCNGNATACCTGTC
CNNCTTNCTCNNNNNGNNNCGTNNNCTTNCTCATAGCTCANGCNNTNGNANNNTCANNNNNNNTNNNNNNNNCNNNNCAG
CTNGGNNTNNNNNNNCNNNNNCCCNNTTCANNNNNNNNTNNCNNTNNNNNCNGNAANNNATCNNNNNGNNNNCNNNNNNN

>colony 5
NNNNNNNNNNNNANNNNGNNNNNGGGCCCGACGTCGCATGCTCCCGGCCGCCATGGCGGCCGCGGGAATTCGATTGGACA
CTGACATGGACTGAAGGAGTAGAAATTCTCTCACATCCACCTGTTCTAAGACTGTAGACGCCATTAACCCTGGGCCTCTA
CCTTACACCAAGTCGAATTCACGACATGCCGTCGGGCCGGCTTTGAACACCAATCTGAAGCCGCGAGCCCGGCAGGGCAG
TGCGAACGATCCTCAGAGCATAGCAGCTCGCCATCGGAGGGAACGCATCAGCGAGCGACTGAAGACTCTACAAGATCTTG
TTCCTAATGGTTCCAAGGTAGACTTGGTCACCATGCTTGAAAAGGCAATCACTAGTGAATTCGCGGCCGCCTGCAGGTCG
ACCATATGGGAGAGCTCCCAACGCGTTGGATGCATAGCTTGAGTATTCTATAGTGTCACCTAAATAGCTTGGCGTAATCA
TGGTCATAGCTGTTTCCTGTGTGAAATTGTTATCCGCTCACAATTCCACACAACATACGAGCCGGAAGCATAAAGTGTAA
AGCCTGGGGTGCCTAATGAGTGAGCTAACTCACATTAATTGCGTTGCGCTCACTGCCCGCTTTCCAGTCGGGAAACCTGT
CGTGCCAGCTGCATTAATGAATCGGCCAACGCGCGGGGAGAGGCGGTTTGCGTATTGGGCGCTCTTCCGCTTCCTCGCTC
ACTGACTCGCTGCGCTCGGTCGTTCGGCTGCGGCGAGCGGTATCAGCTCACTCAAAGGCGGTAATACGGTTATCCACAGA
ATCAGGGGATAACGCANGAAAGAACATGTGAGCAAAAGGCCAGCAAAAGGCCAGGAACCGTAAAAAGGCCGCGTTGCTGG
CGTTTTTCCATAGGCTCCGCCCCCCTGACGAGCATCACAAAAATCGACGCTCAANTCANAGGTGGCGAAACCCGACAGGA
CTATAAAGATACCAGGNGTTTCCCCCNGGNAAGCTCCNCGTGCGCTCTCNGTNNCGACCCTGCNNCTNANCNGNATANCN
GTCNNNNTNNTCCCNNCGGNAGNNNNNNGCNTNCTCANNNCTCANGCNGNAGNNTCTCANNNNNNNNNNGGNCNNNTCGC
NNCNNNNNNGNNNNNNTNNNCNANCCNNNNNNANCCNNNNCNNNNNNNNTNNNNANNNNNTCNNNNTNNANN

>colony 6
NNANCNGNNNNNNNNNNNNNNNNNNTNCNGNNAGGNNNNNNNNNNNTGNNNNNNNGGTTNGNNNNNNNNCNNCCNAGNNNGAGNNNNNNNNNNNNCNNNTGAGANNCTANNGCNTGAGCTATGAGAAAGCNCNCGCTTCCNANGGNAGAAAGNCGGACAGNTNTCNNNTAAGCGGCAGGGTCGGAACNGNGAGCGCACGNGGAGNTTNCCNGGGGGAAACGNNNGGTATCTTTATAGTCCTGTCGGNTTTCGCCACNTCTGACTTGAGCGTCGATTTTTGTGATGCTCGTCAGGGGGGCGGAGCCTATGGAAAAACGCCAGCAACGCGGCCTTTTTACGGTTCCTGGCCTTTTGCTGNCCTTTTGCTCACATGTTCTTTCNTGCGTTATCCCCTGATTCTGTGGATAACCGTATTACCGCCTTTGAGTGAGCTGATACCGCTCGCCGCAGCCGAACGACCGAGCGCAGCGAGTCAGTGAGCGAGGAAGCGGAAGAGCGCCCAATACGCAAACCGCCTCTCCCCGCGCGTTGGCCGATTCATTAATGCAGCTGGCACGACAGGTTTCCCGACTGGAAAGCGGGCAGTGAGCGCAACGCAATTAATGTGAGTTAGCTCACTCATTAGGCACCCCAGGCTTTACACTTTATGCTTCCGGCTCGTATGTTGTGTGGAATTGTGAGCGGATAACAATTTCACACAGGAAACAGCTATGACCATGATTACGCCAAGCTATTTAGGTGACACTATAGAATACTCAAGCTATGCATCCAACGCGTTGGGAGCTCTCCCATATGGTCGACCTGCAGGCGGCCGCGAATTCACTAGTGATTGGACACTGACATGGACTGAAGGAGTAGAAATTCTCTCACATCCACCTGTTCTAAGACTGTAGACGCCATTAACCCTGGGCCTCTACCTTACACCAAGTCGAATTCACGACATGCCGTCGGGCCGGCTTTGAACACCAATCTGAAGCCGCGAGCCCGGCAGGGCAGTGCGAACGATCCTCAGAGCATAGCAGCTCGCCATCGGAGGGAACGCATCAGCGAGCGACTGAAGACTCTACAAGATCTTGTTCCTAATGGTTCCAAGGTAGACTTGGTCACCAAATCGAATTCCCGCGGCCGCCATGGCGGCCGGGAGCATGCGACGTCGGCCCNNNNGCCNNNNNNNNNNNNNN

>colony 7
NNNNNNNNNNNNNANGGCGATTGGGCCCGACGTCGCATGCTCCCGGCCGCCATGGCGGCCGCGGGAATTCGATTGGACAC
TGACATGGACTGAAGGAGTAGAAATTCTCTCACATCCACCTGTTCTAAGACTGTAGACGCCATTAACCCTGGGCCTCTAC
CTTACACCAAGTCGAATTCACGACATGCCGTCGGGCCGGCTTTGAACACCAATCTGAAGCCGCGAGCCCGGCAGGGCAGT
GCGAACGATCCTCAGAGCATAGCAGCTCGCCATCGGAGGGAACGCATCAGCGAGCGACTGAAGACTCTACAAGATCTTGT
TCCTAATGGTTCCAAGGTAGACTTGGTCACCATGCTTGAAAAGGCAATCACTAGTGAATTCGCGGCCGCCTGCAGGTCGA
CCATATGGGAGAGCTCCCAACGCGTTGGATGCATAGCTTGAGTATTCTATAGTGTCACCTAAATAGCTTGGCGTAATCAT
GGTCATAGCTGTTTCCTGTGTGAAATTGTTATCCGCTCACAATTCCACACAACATACGAGCCGGAAGCATAAAGTGTAAA
GCCTGGGGTGCCTAATGAGTGAGCTAACTCACATTAATTGCGTTGCGCTCACTGCCCGCTTTCCAGTCGGGAAACCTGTC
GTGCCAGCTGCATTAATGAATCGGCCAACGCGCGGGGAGAGGCGGTTTGCGTATTGGGCGCTCTTCCGCTTCCTCGCTCA
CTGACTCGCTGCGCTCGGTCGTTCGGCTGCGGCGAGCGGTATCAGCTCACTCAAAGGCGGTAATACGGTTATCCACAGAA
TCANGGGATAACGCNNAAAGAACATGTGAGCAAAAGGCCAGCAAAAGGCCAGGAACCGTAAAAAGGCCGCGTTGCTGGCG
TTTTTCCATAGGCTCCGCCCCCCTGACGAGCATCACAAAAATCGACGCTCAAGTCAGAGGTGGCGAAANCCGACAGGNAC
TATAAAGATACCNGGCGTTTCCCCCNGGNNGCTCCCNCGNGCGCNNNTCCTGNNCNACCNNNNNTACNNATNCNNNCCGN
CNTNCTCCNNCGGGAANCNNGNNGCTTNNNATAGCTCANGCNNNAGNATCNNCANNNNNNTANGNCNNNCNNNNNNNNTN
GGNNNNNNNNNNNNNCNCNNNNANNNNNNNNNNNNNNNGNANNNNNNNNNNNNNNNNNNNNTAGNN

>colony 8
NNNNNNNNNNNNNNGGNNNTTGGGNNNACGTCGCATGCTCCCGGCCGCCATGGCGGCCGCGGGAATTCGATTGGACACTG
ACATGGACTGAAGGAGTAGAAATTCTCTCACATCCACCTGTTCTAAGACTGTAGACGCCATTAACCCTGGGCCTCTACCT
TACACCAAGTCGAATTCACGACATGCCGTCGGGCCGGCTTTGAACACCAATCTGAAGCCGCGAGCCCGGCAGGGCAGTGC
GAACGATCCTCAGAGCATAGCAGCTCGCCATCGGAGGGAACGCATCAGCGAGCGACTGAAGACTCTACAAGATCTTGTTC
CTAATGGTTCCAAGGTAGACTTGGTCACCATGCTTGAAAAGGCAATCACTAGTGAATTCGCGGCCGCCTGCAGGTCGACC
ATATGGGAGAGCTCCCAACGCGTTGGATGCATAGCTTGAGTATTCTATAGTGTCACCTAAATAGCTTGGCGTAATCATGG
TCATAGCTGTTTCCTGTGTGAAATTGTTATCCGCTCACAATTCCACACAACATACGAGCCGGAAGCATAAAGTGTAAAGC
CTGGGGTGCCTAATGAGTGAGCTAACTCACATTAATTGCGTTGCGCTCACTGCCCGCTTTCCAGTCGGGAAACCTGTCGT
GCCAGCTGCATTAATGAATCGGCCAACGCGCGGGGAGAGGCGGTTTGCGTATTGGGCGCTCTTCCGCTTCCTCGCTCACT
GACTCGCTGCGCTCGGTCGTTCGGCTGCGGCGAGCGGTATCAGCTCACTCAAAGGCGGTAATACGGTTATCCACAGAATC
AGGGGATAACGCANGAAAGAACATGTGAGCAAAANGCCAGCAAAAGGCCAGGAACCGTAAAAAGGCCGCGTTGCTGGCGT
TTTTCCATAGGCTCCGCCCCCCTGACGAGCATCACAAAAATCGACGCTCAAGTCAGANGTGGCGAAANCCGACAGGNCTA
TAAAGATACCAGGCGTTTCCCCCNNGNAGCTCCCNCGTGCGCTCTNCTGNTTCCGACCCNGCCGNTTACCNGNATACCTG
TCCNCCNTTCTCCCNNCGGNAGCGTNNNCTTTCTCATAGCNCNNNGCTNNANGNNNNTCANTNNNGNGTAGNNNNNNNNN
NNCNNCTGNNNNGGNNNNNNCNNNNCCCCNNNCANCCNGNNNNNNNNNCTNATNNNNNNCNATCNNNNNNNNNNNNNNNN
CGNNNNNNNNNN

>colony 9
NNNNNNNNNNNANNGGGGCGATTGGGCCCGACGTCGCATGCTCCCGGCCGCCATGGCGGCCGCGGGAATTCGATTGGACA
CTGACATGGACTGAAGGAGTAGAAATTCTCTCACATCCACCTGTTCTAAGACTGTAGACGCCATTAACCCTGGGCCTCTA
CCTTACACCAAGTCGAATTCACGACATGCCGTCGGGCCGGCTTTGAACACCAATCTGAAGCCGCGAGCCCGGCAGGGCAG
TGCGAACGATCCTCAGAGCATAGCAGCTCGCCATCGGAGGGAACGCATCAGCGAGCGACTGAAGACTCTACAAGATCTTG
TTCCTAATGGTTCCAAGGTAGACTTGGTCACCATGCTTGAAAAGGCAATCACTAGTGAATTCGCGGCCGCCTGCAGGTCG
ACCATATGGGAGAGCTCCCAACGCGTTGGATGCATAGCTTGAGTATTCTATAGTGTCACCTAAATAGCTTGGCGTAATCA
TGGTCATAGCTGTTTCCTGTGTGAAATTGTTATCCGCTCACAATTCCACACAACATACGAGCCGGAAGCATAAAGTGTAA
AGCCTGGGGTGCCTAATGAGTGAGCTAACTCACATTAATTGCGTTGCGCTCACTGCCCGCTTTCCAGTCGGGAAACCTGT
CGTGCCAGCTGCATTAATGAATCGGCCAACGCGCGGGGAGAGGCGGTTTGCGTATTGGGCGCTCTTCCGCTTCCTCGCTC
ACTGACTCGCTGCGCTCGGTCGTTCGGCTGCGGCGAGCGGTATCAGCTCACTCAAAGGCGGTAATACGGTTATCCACAGA
ATCAGGGGATAACGCANGAAAGAACATGTGAGCAAAANGNCAGCAAAAGGCCAGGAACCGTAAAAAGGCCGCGTTGCTGG
CGTTTTTCCATAGGCTCCGCCCCCCTGACGAGCATCACAAAAATCGACGCTCAAGTCAGANGTGGCGAANNCCGACAGGA
CTATAAAGATACCAGGNGTTTCCCCCNNNNNGCTTCCCTCGTGCGCTCNNCNGTNCCGANNNNCCGCTTANCNNATACCT
NNCCGCCTTTNNTCCCNNCGGAAGCNNNNNCTTTNNTCATANNNNACGCNNNNGNANNNCANTTNNNNNNNGNCNNNNNC
NNNNNNNNNNNGNNNNNNNCNNNNCCCNNNNCNNNCNNNNCNNNNCTATNNNGNANNNNNNNNNNNGANNNNNNNNNNNG
NNNNNNNNNNN

>colony 10
NNNNNNNNNNNTNNNGCGANTGGGCCCGACGTCGCATGCTCCCGGCCGCCATGGCGGCCGCGGGAATTCGATTGGACACT
GACATGGACTGAAGGAGTAGAAATTCTCTCACATCCACCTGTTCTAAGACTGTAGACGCCATTAACCCTGGGCCTCTACC
TTACACCAAGTCGAATTCACGACATGCCGTCGGGCCGGCTTTGAACACCAATCTGAAGCCGCGAGCCCGGCAGGGCAGTG
CGAACGATCCTCAGAGCATAGCAGCTCGCCATCGGAGGGAACGCATCAGCGAGCGACTGAAGACTCTACAAGATCTTGTT
CCTAATGGTTCCAAGGTAGACTTGGTCACCATGCTTGAAAAGGCAAATCACTAGTGAATTCGCGGCCGCCTGCAGGTCGA
CCATATGGGAGAGCTCCCAACGCGTTGGATGCATAGCTTGAGTATTCTATAGTGTCACCTAAATAGCTTGGCGTAATCAT
GGTCATAGCTGTTTCCTGTGTGAAATTGTTATCCGCTCACAATTCCACACAACATACGAGCCGGAAGCATAAAGTGTAAA
GCCTGGGGTGCCTAATGAGTGAGCTAACTCACATTAATTGCGTTGCGCTCACTGCCCGCTTTCCAGTCGGGAAACCTGTC
GTGCCAGCTGCATTAATGAATCGGCCAACGCGCGGGGAGAGGCGGTTTGCGTATTGGGCGCTCTTCCGCTTCCTCGCTCA
CTGACTCGCTGCGCTCGGTCGTTCGGCTGCGGCGAGCGGTATCAGCTCACTCAAAGGCGGTAATACGGTTATCCACAGAA
TCNNGGGATAACGCANGAAAGAACATGTGAGCAAAAGGCCAGCAAAAGGCCAGGAACCGTAAAAAGGCCGCGTTGCTGGC
GTTTTTCCATAGGCTCCGCCCCCCTGACGAGCATCACAAAAATCGACGCTCAAGTCAGANNTGGNGAAANCCGACAGGAC
TATAAAGATACCAGNNTTTNCCCCTNNAGCTCCNTCGTGCGNTNNNCCTGTTCCGACCNNCNCTTACNNNANNNNTGTCN
NNNNNTNTCNNNNNNNNNNNGNCGNTTTNNCNNAGCNNANNCNNNAGNNNNNNCANNNNNNNANGNCNTNNNNNNCNNNN
NNNNNGNNNNNNNNNCNCCNNNNANCCNNNCNNNNNNNNNGNANNNNNNNNNGNNCNNNNCNNNAGANNNNNNN

>colony 11
NNNNNNNNNNNANGGCNNTTGGGNNNACGTCGCATGCTCCCGGCCGCCATGGCGGCCGCGGGAATTCGATTGGACACTGA
CATGGACTGAAGGAGTAGAAATTCTCTCACATCCACCTGTTCTAAGACTGTAGACGCCATTAACCCTGGGCCTCTACCTT
ACACCAAGTCGAATTCGCGACATGCCGTCGGGCCGGCTTTGAACACCAATCTGAAGCCGCGAGCCCGGCAGGGCAGTGCG
AACGATCCTCAGAGCATAGCAGCTCGCCATCGGAGGGAACGCATCAGCGAGCGACTGAAGACTCTACAAGATCTTGTTCC
TAATGGTTCCAAGGTAGACTTGGTCACCATGCTTGAAAAGGCAATCACTAGTGAATTCGCGGCCGCCTGCAGGTCGACCA
TATGGGAGAGCTCCCAACGCGTTGGATGCATAGCTTGAGTATTCTATAGTGTCACCTAAATAGCTTGGCGTAATCATGGT
CATAGCTGTTTCCTGTGTGAAATTGTTATCCGCTCACAATTCCACACAACATACGAGCCGGAAGCATAAAGTGTAAAGCC
TGGGGTGCCTAATGAGTGAGCTAACTCACATTAATTGCGTTGCGCTCACTGCCCGCTTTCCAGTCGGGAAACCTGTCGTG
CCAGCTGCATTAATGAATCGGCCAACGCGCGGGGAGAGGCGGTTTGCGTATTGGGCGCTCTTCCGCTTCCTCGCTCACTG
ACTCGCTGCGCTCGGTCGTTCGGCTGCGGCGAGCGGTATCAGCTCACTCAAAGGCGGTAATACGGTTATCCACAGAATCA
GGGGATAACGCANGAAAGAACATGTGAGCAAAAGGNCAGCAAAAGGCCAGGAACCGTAAAAAGGCCGCGTTGCTGGCGTT
TTTCCATAGGCTCCGCCCCCCTGACGAGCATCACAAAAATCGACGCTCAAGTCAGAGGTGGCGAAACCCGACAGGACTAT
AAAGATACCAGGCGTTTCCCCCNGNNNGCTCCCTCNTGCGCTCTCCTNNNCGACCCNGCCGCTTANCNGNATACCTGTCC
GCCNTTCTCCCNNNNNNNCGNNNNNTTNCTCANAGCTNANNNNGTAGNNNNNTCANNNGNNNNANGNNCNNNNCNNCNNC
TGGNNNNNNNNNNNNNNCCCCCNNNCANNNNNNNCNNNNNNNTNNNNNNTATCNNNNNNNNNCCNNNNNNNNNN

>colony 12
NNNNNNNNNNNTNNGGCNANTGGGCCNGACGTCGCATGCTCCCGGCCGCCATGGCGGCCGCGGGAATTCGATTGGACACT
GACATGGACTGAAGGAGTAGAAATTCTCTCACATCCACCTGTTCTAAGACTGTAGACGCCATTAACCCTGGGCCTCTACC
TTACACCAAGTCGAATTCACGACATGCCGTCGGGCCGGCTTTGAACACCAATCTGAAGCCGCGAGCCCGGCAGGGCAGTG
CGAACGATCCTCAGAGCATAGCAGCTCGCCATCGGAGGGAACGCATCAGCGAGCGACTGAAGACTCTACAAGATCTTGTT
CCTAATGGTTCCAAGGTAGACTTGGTCACCATGCTTGAAAAGGCAATCACTAGTGAATTCGCGGCCGCCTGCAGGTCGAC
CATATGGGAGAGCTCCCAACGCGTTGGATGCATAGCTTGAGTATTCTATAGTGTCACCTAAATAGCTTGGCGTAATCATG
GTCATAGCTGTTTCCTGTGTGAAATTGTTATCCGCTCACAATTCCACACAACATACGAGCCGGAAGCATAAAGTGTAAAG
CCTGGGGTGCCTAATGAGTGAGCTAACTCACATTAATTGCGTTGCGCTCACTGCCCGCTTTCCAGTCGGGAAACCTGTCG
TGCCAGCTGCATTAATGAATCGGCCAACGCGCGGGGAGAGGCGGTTTGCGTATTGGGCGCTCTTCCGCTTCCTCGCTCAC
TGACTCGCTGCGCTCGGTCGTTCGGCTGCGGCGAGCGGTATCAGCTCACTCAAAGGCGGTAATACGGTTATCCACAGAAT
CANGGGATAACGCANGAAAGAACATGTGAGCAAAAGGCCAGCAAAAGGCCAGGAACCGTAAAAAGGCCGCGTTGCTGGCG
TTTTTCCATAGGCTCCGCCCCCCTGACGAGCATCACAAAAATCGACGCTCAAGTCAGAGGTGGCGAANCCGACAGGACTA
TAAAGATACCAGGCGTTNCCCCCTGNNNGCTCCCTCNNGCNCTNNNNNGTTCCGAACNNNNCGCTTACCNGNATNCCTNN
NTCCGCNTTTCTCCCNTTCGNNNNNNGNNNCTTTNNNCNTANCTNCACGCTGTAGNATCTCAGTTNNNNNNGNNNNNNNN
NNCNNNNNNGGNNNNNNNNNNNNNCCCNCCNNTTCANNCCNNACNCNNNNNCTNNNNCNGNNANCNNNCGTNNTNNNNNN
NNNNCCNNNGNTNANANNNNNNNN
