# Supplementary material for: Negative regulation of conserved RSL class I bHLH transcription factors evolved independently among land plants
Source: eLife. 2018 Aug 23;7:e38529. doi: 10.7554/eLife.38529 (PMC6141232; doi:10.7554/eLife.38529)
Supplement: Supplementary file 4. — polymorpha and their orthologs from other liverworts. The predicted MpFRH1 target site is indicated with a grey arrow. Region around the predicted miRNA target site (top) and overview of the alignment (bottom). (A) Foie gras domain containing protein Mapoly0075s0041.1. (B) Basic helix-loop-helix transcription factor MpRSL1 Mapoly0039s003 (C) Basic helix-loop-helix transcription factor transcript 27676. (D) Nucleotide-rhamnose synthase/epimerase-reductase Mapoly0005s0120. [file elife-38529-supp4.pdf]

Supplemental file 4. Multiple sequence alignments of the predicted MpFRH1 miRNA target mRNAs in *M. polymorpha* and their orthologs from other liverworts. The predicted MpFRH1 target site is indicated with a grey arrow. Region around the predicted miRNA target site (top) and overview of the alignment (bottom). A) Foie gras domain containing protein Mapoly0075s0041.1. B) Basic helix-loop-helix transcription factor MpRSL1 Mapoly0039s003 C) Basic helix-loop-helix transcription factor transcript 27676  D) Nucleotide-rhamnose synthase/epimerase-reductase Mapoly0005s0120.

A

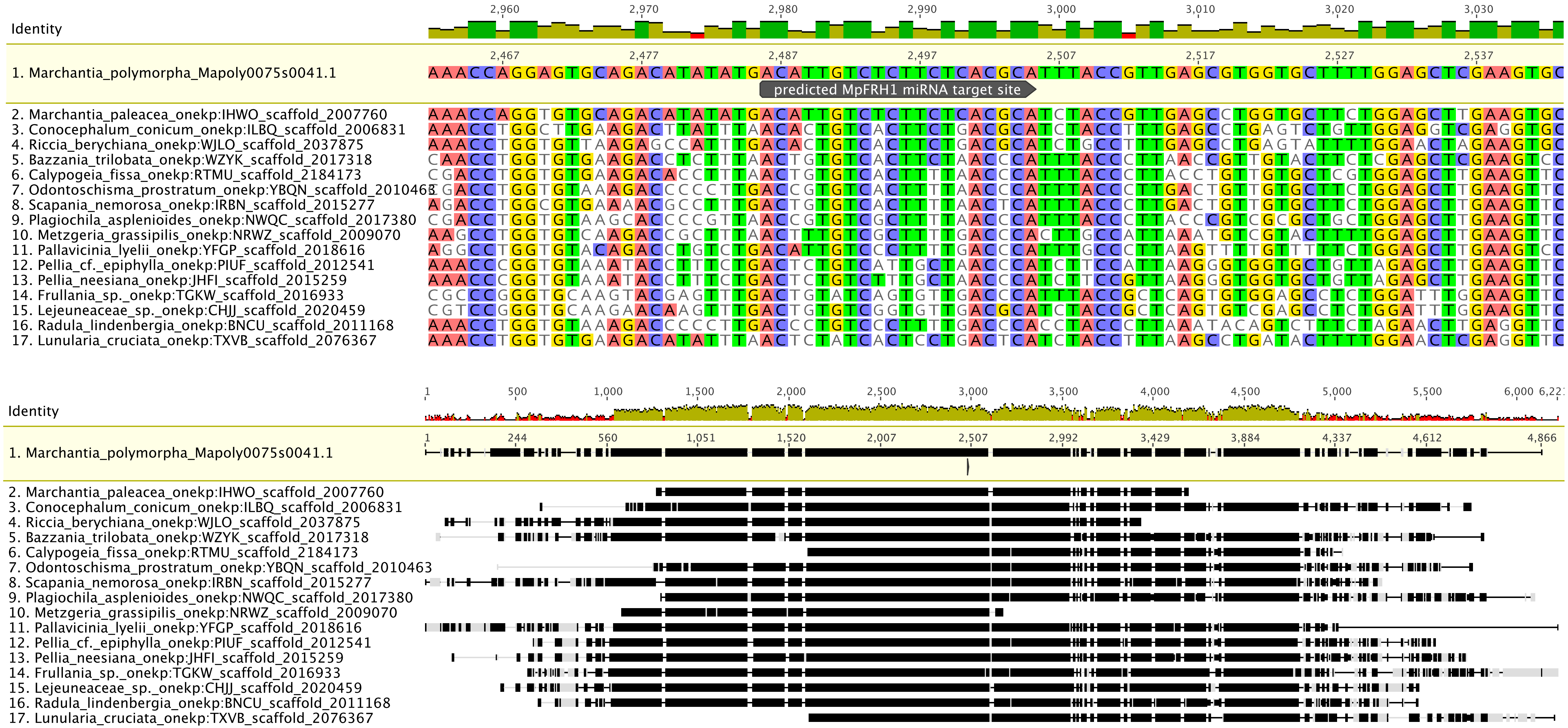

B

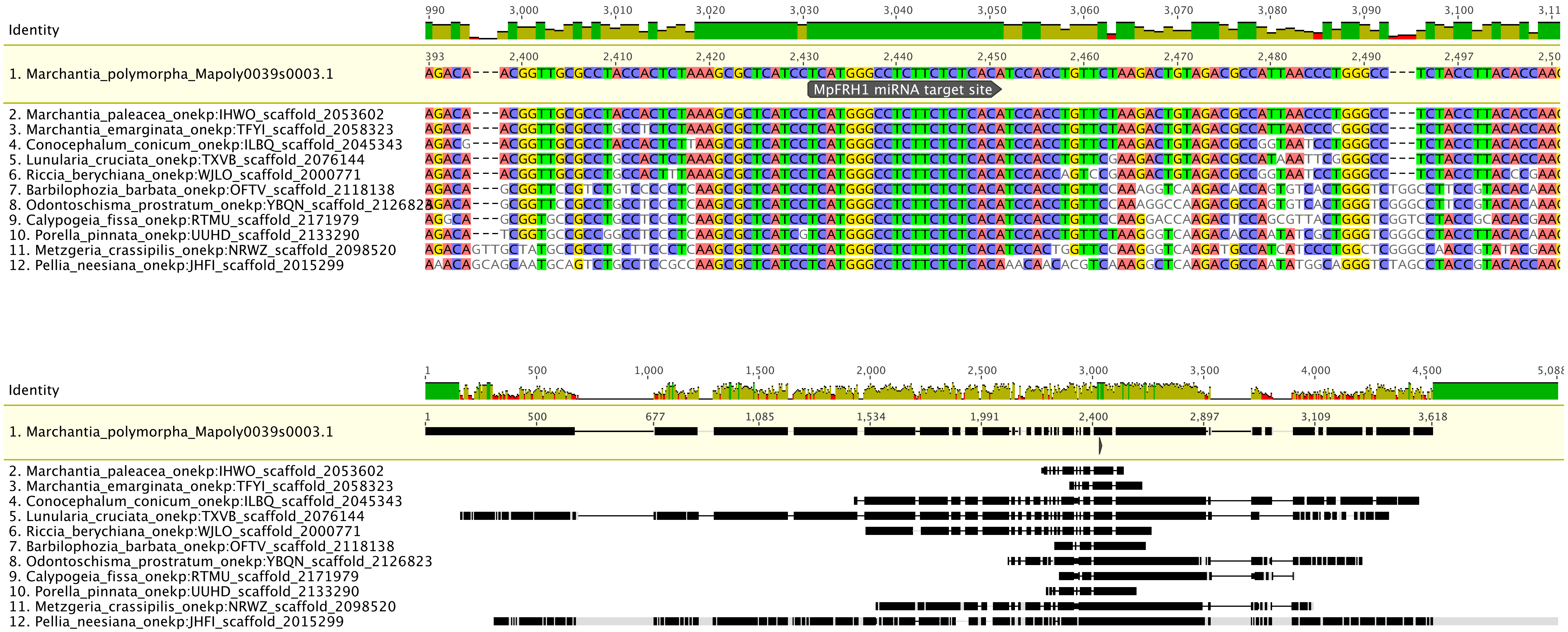
Identity
1. Marchantia_polymorpha_Mapoly0039s0003.1
MpFRH1 miRNA target site
2. Marchantia_paleacea_onekp:IHWO_scaffold_2053602
3. Marchantia_emarginata_onekp:TFYI_scaffold_2058323
4. Conocephalum_conicum_onekp:ILBQ_scaffold_2045343
5. Lunularia_cruciata_onekp:TXVB_scaffold_2076144
6. Riccia_berychiana_onekp:WJLO_scaffold_2000771
7. Barbilophozia_barbata_onekp:OFTV_scaffold_2118138
8. Odontoschisma_prostratum_onekp:YBQN_scaffold_2126823
9. Calypogeia_fissa_onekp:RTMU_scaffold_2171979
10. Porella_pinnata_onekp:UUHD_scaffold_2133290
11. Metzgeria_crassipilis_onekp:NRWZ_scaffold_2098520
12. Pellia_neesiana_onekp:JHFI_scaffold_2015299

C

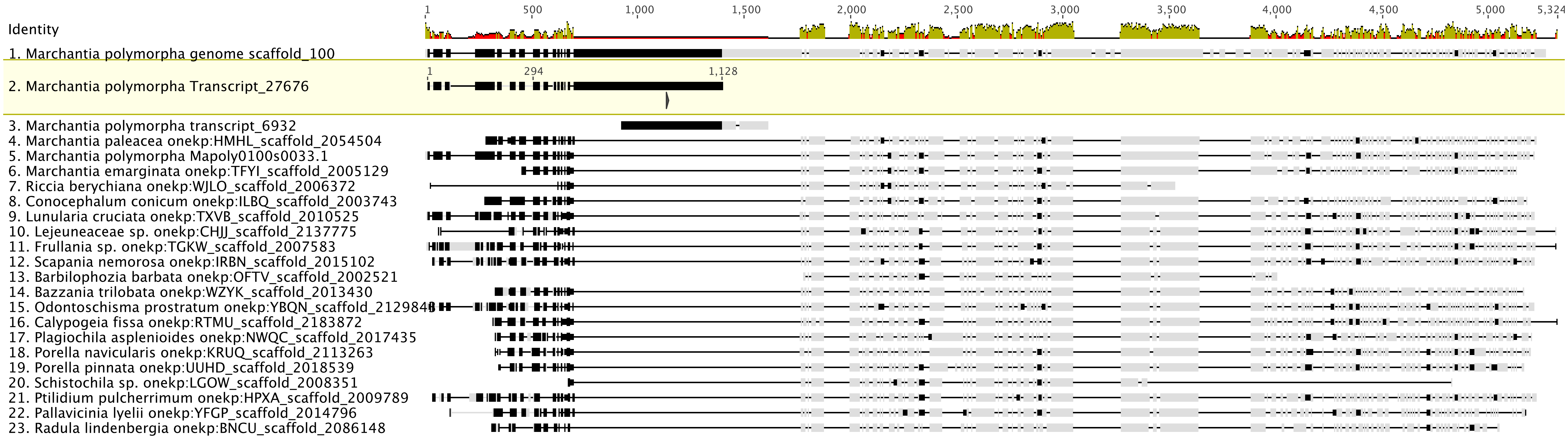

1
500
1,000
1,500
2,000
2,500
3,000
3,500
4,000
4,500
5,000
5,324
Identity
1. Marchantia polymorpha genome scaffold_100
1
294
1,128
2. Marchantia polymorpha Transcript_27676
3. Marchantia polymorpha transcript_6932
4. Marchantia paleacea onekp:HMHL_scaffold_2054504
5. Marchantia polymorpha Mapoly0100s0033.1
6. Marchantia emarginata onekp:TFYI_scaffold_2005129
7. Riccia berychiana onekp:WJLO_scaffold_2006372
8. Conocephalum conicum onekp:ILBQ_scaffold_2003743
9. Lunularia cruciata onekp:TXVB_scaffold_2010525
10. Lejeuneaceae sp. onekp:CHJJ_scaffold_2137775
11. Frullania sp. onekp:TGKW_scaffold_2007583
12. Scapania nemorosa onekp:IRBN_scaffold_2015102
13. Barbilophozia barbata onekp:OFTV_scaffold_2002521
14. Bazzania trilobata onekp:WZYK_scaffold_2013430
15. Odontoschisma prostratum onekp:YBQN_scaffold_212984
16. Calypogeia fissa onekp:RTMU_scaffold_2183872
17. Plagiochila asplenioides onekp:NWQC_scaffold_2017435
18. Porella navicularis onekp:KRUQ_scaffold_2113263
19. Porella pinnata onekp:UUHD_scaffold_2018539
20. Schistochila sp. onekp:LGOW_scaffold_2008351
21. Ptilidium pulcherrimum onekp:HPXA_scaffold_2009789
22. Pallavicinia lyelii onekp:YFGP_scaffold_2014796
23. Radula lindenbergia onekp:BNCU_scaffold_2086148

D

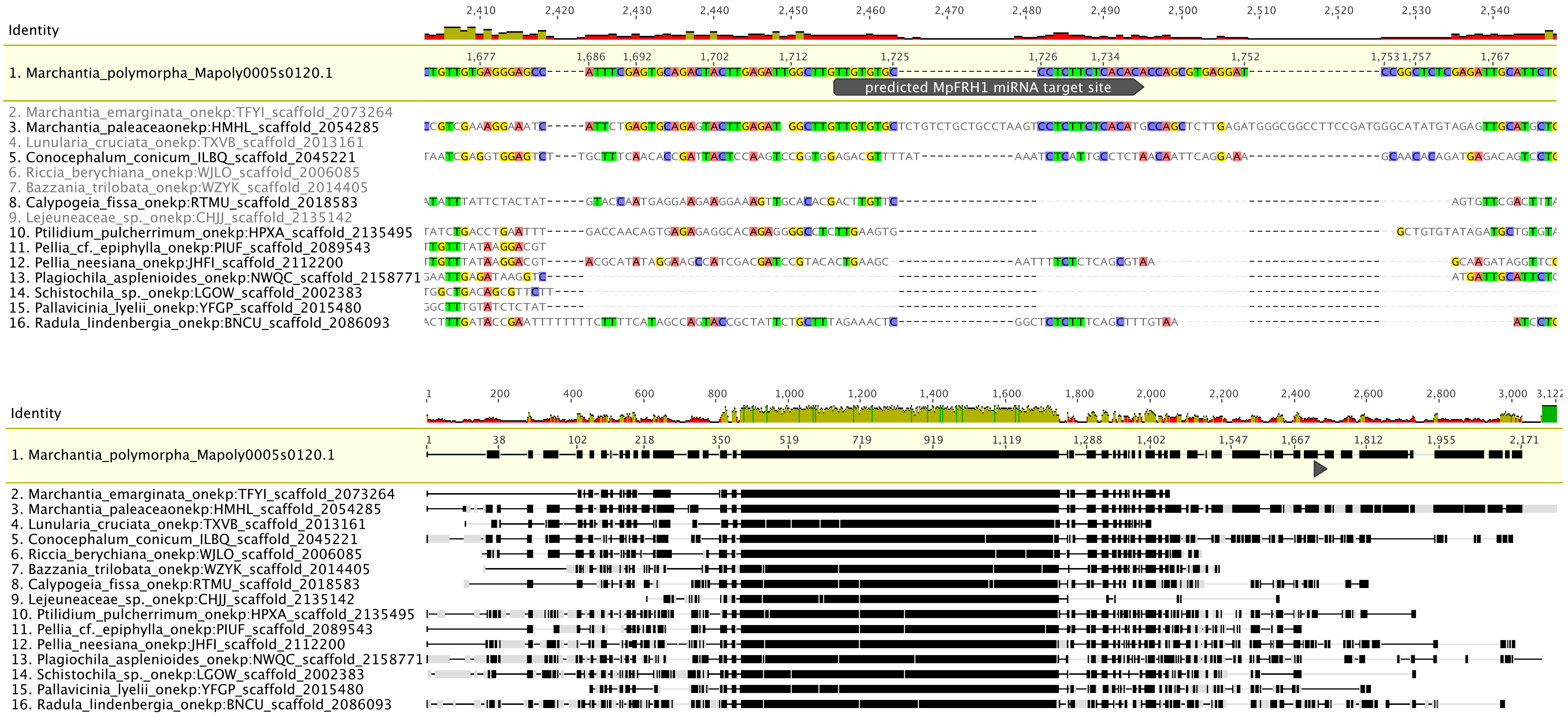

Identity
2,410
2,420
2,430
2,440
2,450
2,460
2,470
2,480
2,490
2,500
2,510
2,520
2,530
2,540
1. Marchantia_polymorpha_Mapoly0005s0120.1
predicted MpFRH1 miRNA target site
2. Marchantia_emarginata_onekp:TFYI_scaffold_2073264
3. Marchantia_paleaceaonekp:HMHL_scaffold_2054285
4. Lunularia_cruciata_onekp:TXVB_scaffold_2013161
5. Conocephalum_conicum_ILBQ_scaffold_2045221
6. Riccia_berychiana_onekp:WJLO_scaffold_2006085
7. Bazzania_trilobata_onekp:WZYK_scaffold_2014405
8. Calypogeia_fissa_onekp:RTMU_scaffold_2018583
9. Lejeuneaceae_sp._onekp:CHJJ_scaffold_2135142
10. Ptilidium_pulcherrimum_onekp:HPXA_scaffold_2135495
11. Pellia_cf._epiphylla_onekp:PIUF_scaffold_2089543
12. Pellia_neesiana_onekp:JHFI_scaffold_2112200
13. Plagiochila_asplenioides_onekp:NWQC_scaffold_2158771
14. Schistochila_sp._onekp:LGOW_scaffold_2002383
15. Pallavicinia_lyelii_onekp:YFGP_scaffold_2015480
16. Radula_lindenbergia_onekp:BNCU_scaffold_2086093
Identity
1
200
400
600
800
1,000
1,200
1,400
1,600
1,800
2,000
2,200
2,400
2,600
2,800
3,000
1. Marchantia_polymorpha_Mapoly0005s0120.1
2. Marchantia_emarginata_onekp:TFYI_scaffold_2073264
3. Marchantia_paleaceaonekp:HMHL_scaffold_2054285
4. Lunularia_cruciata_onekp:TXVB_scaffold_2013161
5. Conocephalum_conicum_ILBQ_scaffold_2045221
6. Riccia_berychiana_onekp:WJLO_scaffold_2006085
7. Bazzania_trilobata_onekp:WZYK_scaffold_2014405
8. Calypogeia_fissa_onekp:RTMU_scaffold_2018583
9. Lejeuneaceae_sp._onekp:CHJJ_scaffold_2135142
10. Ptilidium_pulcherrimum_onekp:HPXA_scaffold_2135495
11. Pellia_cf._epiphylla_onekp:PIUF_scaffold_2089543
12. Pellia_neesiana_onekp:JHFI_scaffold_2112200
13. Plagiochila_asplenioides_onekp:NWQC_scaffold_2158771
14. Schistochila_sp._onekp:LGOW_scaffold_2002383
15. Pallavicinia_lyelii_onekp:YFGP_scaffold_2015480
16. Radula_lindenbergia_onekp:BNCU_scaffold_2086093
