# Supplementary material for: Negative regulation of conserved RSL class I bHLH transcription factors evolved independently among land plants
Source: eLife. 2018 Aug 23;7:e38529. doi: 10.7554/eLife.38529 (PMC6141232; doi:10.7554/eLife.38529)
Supplement: Supplementary file 5. [file elife-38529-supp5.docx]

**Supplemental file 5: promoter analysis**

Mp*FRH1* promoter construct:

3.5 kb region immediately 5’ of the Mp*FRH1* transcript producing genomic region was cloned as the Mp*FRH1* promoter. This corresponds to scaffold_43:382450-378958 in the *M. polymorpha* genome sequence published in Bowman et al., 2017.

>ProMp*FRH1*

ATTTAAATGAAATCTGAGTTTCCTGTCGATTCAATCTCTCATTGTGTATCATAAACCATACTTCGCAATCTGCGAATGTTCGGGTAAAGCTTTGCGCATTTTTTATTTCCTCACCGAGAATCTTTTGCTCTGCCTTGACCTCACTTTCTTAAAATTCCGTCTGTGGAAGAAAAGCTAATCTGAAAGGCTATAATTTGGTCATTGAAGATAACCTTGATAATATAGGAAACTCGCTGTCAGGGTTGTAAAGAGCCGTGAAACAAAACAGTGACGAGAGAGCTACCTGAAGTTTCCTACAAAGGGAGTTGATTTGAACGACGGAAACAAATGTTATGTGCTCTTGTGAGTGGAAGCAGAAAGAAACAAGCATCAGAGAGGTTTCCATCACAATCTTGACAGCTGAAGTAACAAGTCGACGATAGAAACATCAGGGAAACTAACGGAAGAGGAAGAAAACCGTTTCTGGGACAACAGTTTGACGACATAGTTCCAATAAGACCGATAGCTCGTACTCCATAATCAGTTCTTCTGCCGTATAAAGTCAGCAACATTGCTATCATAACATGGAAGAAAACTTGTAGCATGTACTTGTTCATTCTGATTTGCGTCTGTTGAAATTTGCATTGATTGCACAATGTTGGACAACGAAGAAACAAATCTCTTGCAGAACCTTCTTCCTCATCATTTTCACGAAAGTGGACGCAATTTAAACCGTCGGCACTGGACTTTCAAAGCAACTTGGACCTTAGCTTACCTCCTTCTCGCCAGTAAATTCGTAGATTGCGTACAGAAAGTCGAGCAGTCACATTTTGCCCTCAACAAAATCTTGTTCTTCAAGCAGTTAGAACCATCGAATTTCTATCAGTGCTCTTGCATCTACTCTAGCCTGGAGCTGCCTCTGTGGATCAGATGGTCCGCAGTTGAGACACAGCCGCTTCACACCTGGAAATTAATTTAATGTGAGAGAGAACCTTCAGTATCAAGCGCAATCTGTCATTTCTACTACGACCCAAGACGTAATACAAACCTCTTTCTCCAATCGGCCATACCGAACACGGCAGATCCATTTGATTGGATCAATAAGTCAAAGGAAAGAGAGAAACGTCATTCATTCAAAGGTTTGAACAGAATGATAATCATATCTAGCGTCTGAATTTTGATATAGAGGTACTTACTTTCTAATCCGATAAGTTTTGAGTGATTCTGATCTCGGGGCGCCCGGTGATGTCAGAAGGGAAGCCAAGATTGAGCAGCAAATTTCCGGAGATTAACAAAATGCTAGAGCTTATCGGTGTTACTTTATGTCTGGCTGGGTCTATGAAACCTACAGGGGCTCTGGGTTACTAGAGTCAGACACGAGGAGCTTCACGAGGAACTACTGACCGGCGAATACCGTGGACAGAAGATACCGTATGAGACCGACAAGAGGTCGTGAGGCATGAATCAAATCTGGAGAAAGCTTTTCAAGGCACGAATGGACCCGATGCACACTTAGAGCGATTTGGAGGATTGGAGCCAGCGAGCCGACGACTGAAGTTGCGCCTGTTTAAATGTGCCGTCTCGAGTAACTTTGGGAAAATCTGGACATAATGCGTCGCAGAGTTGCACGTGAAAGCTGCGGCCGGATCAGAGCTGTCATCTGAATATTCAAAACCTAAGAATCTGTGGGGGACGAGCTCGTGAAGCCTCAGGGCCGGGCGCGTGAGATTATGCTGGCGTCACTTTTTGCAGAAGCCAGTCAGGGTGGGAGTTAAGTCGAAGAGCCGAGAATGATATTCCAAATGCGAGATCCGATGTCGAGAGATCGTGACGGCGAAAGGCAGGATGAGATTGTTATGGCATCATCCTCTGCATTCGCGAAGCACAGGTGCGTATTAAGTCGGAAAGCAGGAGAATGACGCGCAATTGTTCCAGCATCCGGTTGTTGTCAACTTATTACTGCTCGAGGACAGCCACGGACACGATCGTGGAACCCGGGGCAGCCCTAGCGTACGAGAAGTCCGTTTTCAAGCAGAGGCATAAACCTCAAGATCTGCTGCTAGTAAGTTCAGGCCTTATATATCGATCATGAAGAACGTGACATTTGAGGTGCAACATGTGACAGCCTTGAATTACTCAGACATGATCTGCAGCAGTGGCAGACCTGTTGTCTAGAGAATGGCCGTGAAAGCACGATAGGCGAGCACGTCAGACTTGCCAGATTTTCCAAGCGCAGGGCAGCGAGCTCGGAGGACCCGTTTTTCCAGTGCAAGCACATTGAAGCTCTGGGTCGTAGAGAGGCGCTCCATTATCGTTGCGAAATACACATTAATGCAAATGAAAGGACACCTTTCTCGACTCGCACATCGTGACATATGAATTTAGGTCGACATGTGAGAACGTGATCTTGAGACCGAAAACTCTAATTATGTGCCCACACAGTCGACTCCCCTTCACCGTCGTAGGCTGCCTGTGTGATTCCGCCTGACTTAGCCCCGAGCGCTGCTGCAACGTGAGCGTTTTAGTGCAAATCTCTGCCACTAATGATCCACACAAGGACAGAACGTGAGATATGAAGCACGACATGTCCGAACGTGAGATCCCCGTCACGGAAAAAGGAATCGACCTTGTGCCACCCTCTCCCTGGATTGATCCAGGTTCATCAATCCCACCCATAAGCCCAATCACGGGCTCCGCTTCACATCGTCAATCCTCTCTCTCTCTCTCTCTCTCTCTAGAACTGCGTGCATCTTTCTTTCCCTTCTGAATCTACAATTCTCTGTGTCTGTAGGACAAGAGCATTTCTCACTCTACTGACTGACTGGCGCTGCTGCGGCGCGGTGGTCCGGTCCCGGTCAAGTCAAAGGCAGTCGTGTCCGGTGAAGTGCAGTGTCCGCTGTCTAATTCGTCGGCACGAACCGACCGCCCCCGGAGCTAATAATCGTCGCAGGTCGCAGGGAGAAGGTCGCAGGCGCGCGCAGTCGTCACAGTCGGGGAGGGGAAGAGAGAGCGCAGCGCAGCGGCTGAGAGGGGAGGGGAACTAAGAGCTGAGAGCTGAGAGATTTTCACGTTCTGGAACACGACGGGCGCGCAATCCACGATAGATCCGCGCTGGCAAAAGGTGGCCAGGCGAGCAGGGGGCACATGGAGGGGGAAAATCGGAAAATCGCGGTCCCATAAGCAGGGAAGGGTTAAGATTAGGGGCCGGGTTAGGGCTAAATATGGGGCGAGGGGAACAGAGTGGCGCGGGAATGTTTAAGGGGCAAGTTAACAAAACTCCAGGCATTAACGGCAGTAGGCGACGTGGAAGGCTCGGGGAGGCGAACGTGAGGCCTCATAACTGCGCCCGAGTCCCACTTGGCGTTTAATACAGTGGTTGCTGCAGTGCTAAGGCCCTCATCTCCTCCATCACTCCACCATATTTTTCATGCCTCTTCATCCGTGCACTGTGTTCGGCCCTTTGCGCGGACCCGCTCGCTTCCTATATAAATGCCCGCAGGCGCTCTTTCTCCCTCATCATT

Control promoter construct:

Mp*INCOMPLETE ROOTHAIR ELONGATION* (Mp*IRE*) Mapoly0084s0015.1 encodes for a predicted serine-threonine kinase. We previously identified Mp*IRE* in a screen for mutants defective in rhizoid development (Honkanen et al., 2016). The 3.5 kb region 5’ of the Mp*IRE* coding sequence drives ubiquitous 3xYFP-NLS expression in gemmae.


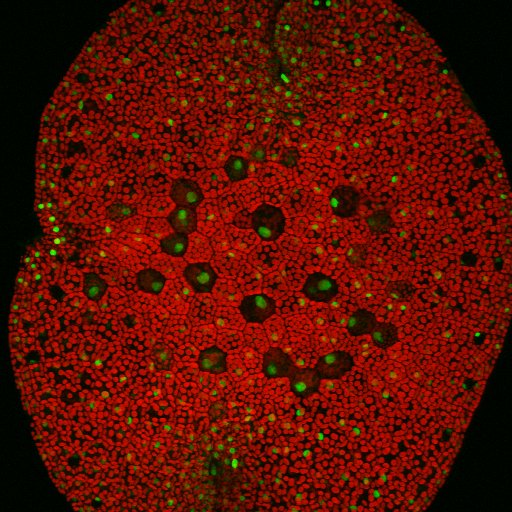
Mp*IRE* promoter expression pattern in one day old gemma.

>proMp*IRE* Mapoly0084s0015.1

AAACAAGATCAGGCTCATCAGACGTGCGAACACGGGGTCAAGGAAGACGCTGGGATCAAGATGACGGGATCGGAACCATGCGGTCGCACTGTGGGCAAAGTCCAAACCGCCGGATCACAGTGAGCAAGGAGATTACGTGGTCAATGTAGCTCGTCAAGCATAAGTAAATCACAATGGAGTGATGGCACTCTATCAACATTAATGGCTGGACATTCCATGGAAGTATAGCATTAAGGTCCATCCACATGCATGAAGTACCCGTATTAATACACGTAGAGCCACGTAGGTAGACATGGGCATCCGGAAACTGGTACGTAGGGGCGAGCGTGTGGTCGATTCTGTTGGATCGGTAATCAGAGAAAATCGGATTGTAAATCCGAACGACTGAATTTCTGCCTCGACTGGAATCACGTCGTCGGCCGACGGGCGGGAGGGAGGGACTACAGGTGCCGCCACCGGCGCGACGCCCTGAATTACCATTGGCCCCCACGAGCCACAATGGGTTATAGGGTGGCATCGGGGGAATTTTATGTTGGCAGATTTTTTCCCCTTTCCTGCCGGGACTTACCGCCGGCACATTTCTTTCCCATCCGCGGAGGCCTTTTCTTTTGGTGACGTGCTCCATCGAGGCGAATTATCCCTCTATGGCTGCAGCAGGAGCTGCCACGTGGCGGCGAGTTGCCGCTTTCCTTCACCGCCCGCTTGCGCGGGCGCGGGTGGTGGGAGCGAGCGGAGCCCTCCTCGTCCTAGTCGGACCTCTCTGCGTTGCCTTGGCCTTTGTTCGGAGCTCCGGGGCAGGGGCAGGGGCAGGGCACGCACGCACGCACTCCTCCGTCCGTTGCTGCCCTGCCTGCCTTCCTTCCTTCCTTCCTTCCTTCCTGCCCGCGCCCGCCCGCCCGCCTTCGCAGTGCACATATGGCAATGTCCATCTGCGTCTGTTTCTCCGGCAATGAGTTCGTTGAGCTGGCACAAGGCAGAGCTGACTGTACCCACCTGACAACCCCATGCCCGCAGATTGACATAAGCCGCATTTCTTTCATGTTCCGTGAGAGGTCGCTCCCGCAGTTTGCCCGGATTTCGCTCGGAAGGACGGTATCCCCGACTGAGGCCCGACCCCTCGACGAGAGAGCGTTCAAATCTTCAATATTTTCCGCGCGGACTTGCGCGGAAAAGAGTTTCTTAGGGTGTCATTGCGGCACAGTAGCCGCGAAGATGGAGTGTCGAAAAATATCCCTCCCCAGACGTAGAACTCACGCTTGCAGAGTGACCTCATTCGCCCGTCATCTTCCAAAGATCCCAGTTCACGTCCGATTTGCAGCGTCGGGTAGTTCGTCCCGCCTGCTGCGAGCGGAGCCCACTGTGCGTCCGGCCGGCCGGACCTCCGGGCGCCTTTGCGTCCTCATCTCCGGCACCGTCATTAACAGGGAAATCGAATCATCAGCCGTACTTGGATGTCATTATTGCGGCGCCGGACTGCCAGAATCCACCTATGGATGCACCGAGCGAGGGAAGGATTTGGCCGCAGCAGCTCCAGCGCCAGTCTCGCAGGGAAAAAGTGCGGCCTGGATCCCGAGCTGTCACCGCAGACGAAAAAGTCCGCCCTGAGCCGCCCCGTGGTGACCTGTGCGTCCTCGGTCGAAACTCAAGACTCGGATTTACGGAAATCCCCCCCGTTCTTGGCTTCCCGTGCGACGGAAGTTAATTATCTCGTCCTTTAAGCCCAGGTCAACGAGTAACTCTTGCCGTCTGGCACGTCCGGCTCTCGATCGGGTTCGCGCGAAGTCCTTCCCTCCCTCAACTGTTGCGCACTGATGGGGGAGCTGGACAGCGGACGCGACCCGACCCGCCAGTGGGCCGTGAAGTGGTGGCGGAATCGGTCGTTTGCGCGTCCCAGAAAGACACGGAGGCCGGAAAGAGCAGGGGGTGCCGGATCCGATCGACAGATCGGAGCGTGTGTGGGTGTGTGTGCGTGCGTGCGTGCGTTCGCAGAGACGGACGGGGAGCCTGGATGGAAATGCCATCGCAGCGTCGCAGGCGGGAGGACGAGGAGGGAGGGGCCGGGCAGGGGACTCGGGAGGGAAGGCGAGGGCAGGGCCCGGCGAGAAAGGGTGAGTTGAGGGTGCTGGCGTTCCTGTTCCGACTGGTCCATTGAGTCTCGGGTCTTCCGATCGCCTCTCTTTCTGCTCCCGCTCGACCTACAGGCAGACAGAGTGACCTATGTAATGTATGTATGCAATTGGCGATTGATCCATCTTCTCCTTTGCCTTCCCGGGCATCGCAGTCGGATGCGCTTTCTTTGCAGCACCACCAAAGCCTTTTTCCGCGCGGGTCCCACTCATTCACTTACAGACGAAATTGTTTGCACAAGTCGGGGACTCGGGGCCGTGCAGGTGAGGAGTTTTTGCGGCAATTCCTCGACAGCCTGCTCTTTCTTTCTCCTTTTTTTTTCGGTTGTTTCTCTGGTGCAAGCGCTGAGAGGGAGCGAGAGAGAAGTAGAGTTGTGGCACACTTGCCCGCCCTGCTGAGAGTGAGTTGCGAGAGGAAGCGAAGACCTAATATACAATCCATCTTTTCGGTTCCGGAGAAAGAAGAGAAGCGAAGCGAGCAGGTTGCAGAGAGGAGAGTGAGGGGGAGATAGAGAGAGGGAGAGAGGGACGGAAAGCAGGAAGGGAGGGCGGGTGTAGGGAGGGGAAGGGAAGGGAAGGGAAGGAAAGGAAAGGGATGAAGAAGAAAATCGATTGAGTGGAAGTGGTTATTTTTTGGGGTCATGAGTTTCCAAGATTCATCCTTTGGAGGAGTACATCTTTTTCTTCAATTGTCGGATTTCATCAAGAAGCCAGTGACAGGGCCTGTGAATCGATAAAGCGCGTGCTGGAGTCAGTGTGTAGGCCATTGTGCTTTGTTGTGTGTAACATGGATTGCCAGGTCTCATCCTCATCGTCATCGTCAGCTAGCTTTGATTTGATAGCGTGATTTTCATGCTATGAACTGCAACTCCTCCTTCGTGTCCATACCCGTATCTGGAGAGGGCGAACTGCAATCTACGTCCCGATGGTAGATAGAAATGCCTGTGATGCCACTTTGCTCCAGAGGGCCCTCTTTAGGCCTGCAGCGACAATCTTCACCTCGTCCCCCGCCAATTATGCGTTGGTATCGCGGAGTGAATTGAAGGAAGTTAATGGTTATCTCAACAGGAATTGGATGTGGCAACCCATTGGATCTGCTGAAACAGAACGCAAAATGGGGAAATTATGGTGAGCCGGCCTTTTGCATGTACCCAGTCTTTCTACCCTTTGCCCACGGATCGAGAGTCGCCAGATGGACGATTACATGTACACTTGTTTCGAAAGTCGATGTTGGATGGATTGTGGACGGACGACTGTTCTTGGCACCTACTAGTTAAACCCTTCCTCTCCTCTTGAACGAAGTACACGCAGAGCATGCTGGGGCGCATCCCTAGATTAGTACATTAGGGAGCGTTCCGTGCACGGTCAATTTTG
