# Supplementary material for: Negative regulation of conserved RSL class I bHLH transcription factors evolved independently among land plants
Source: eLife. 2018 Aug 23;7:e38529. doi: 10.7554/eLife.38529 (PMC6141232; doi:10.7554/eLife.38529)
Supplement: Transparent reporting form [file elife-38529-transrepform.docx]

***eLife’s* transparent reporting form**

We encourage authors to provide detailed information *within their submission* to facilitate the interpretation and replication of experiments. Authors can upload supporting documentation to indicate the use of appropriate reporting guidelines for health-related research (see [EQUATOR Network](http://www.equator-network.org/%20)), life science research (see the [BioSharing Information Resource](https://biosharing.org/)), or the [ARRIVE guidelines](http://www.plosbiology.org/article/info:doi/10.1371/journal.pbio.1000412) for reporting work involving animal research. Where applicable, authors should refer to any relevant reporting standards documents in this form.

If you have any questions, please consult our Journal Policies and/or contact us: [editorial@elifesciences.org](mailto:editorial@elifesciences.org).

**Sample-size estimation**

- You should state whether an appropriate sample size was computed when the study was being designed
- You should state the statistical method of sample size computation and any required assumptions
- If no explicit power analysis was used, you should describe how you decided what sample (replicate) size (number) to use

Please outline where this information can be found within the submission (e.g., sections or figure legends), or explain why this information doesn’t apply to your submission:

Sample size estimations are relevant to qPCR experiments and observation of plant phenotypes. Sample sizes are provided in the relevant sections of materials and methods as well as on the figure legends.

For each qPCR experiment we have included multiple independent transformant lines for each genotype and extracted three biological replicate samples for each line. For observation of plant phenotypes the number of plants observed for each line was generally >100 as indicated in the relevant tables (Figure 1 –figure supplement 1 and Figure 4 –figure supplement 1). An exception is figure 5 –figure supplement 1, where the number of plants observed was limited by the number of transformant lines recovered (as described in the relevant results section) and figure 5, where the number of gemmae observed was limited by the number of gemmae produced by these transformant lines.

**Replicates**

- You should report how often each experiment was performed
- You should include a definition of biological versus technical replication
- The data obtained should be provided and sufficient information should be provided to indicate the number of independent biological and/or technical replicates
- If you encountered any outliers, you should describe how these were handled
- Criteria for exclusion/inclusion of data should be clearly stated
- High-throughput sequence data should be uploaded before submission, with a private link for reviewers provided (these are available from both GEO and ArrayExpress)

Please outline where this information can be found within the submission (e.g., sections or figure legends), or explain why this information doesn’t apply to your submission:

Information regarding the number of biological and technical replicates is relevant to qPCR experiments and observation of plant phenotypes. The information is provided in the relevant methods sections. The number of biological replicates (n) is also indicated in the tables and figure legends.

**Statistical reporting**

- Statistical analysis methods should be described and justified
- Raw data should be presented in figures whenever informative to do so (typically when N per group is less than 10)
- For each experiment, you should identify the statistical tests used, exact values of N, definitions of center, methods of multiple test correction, and dispersion and precision measures (e.g., mean, median, SD, SEM, confidence intervals; and, for the major substantive results, a measure of effect size (e.g., Pearson's r, Cohen's d)
- Report exact p-values wherever possible alongside the summary statistics and 95% confidence intervals. These should be reported for all key questions and not only when the p-value is less than 0.05.

Please outline where this information can be found within the submission (e.g., sections or figure legends), or explain why this information doesn’t apply to your submission:

The statistical analysis methods used are indicated in the figure legends. We chose not to use statistical analysis for qPCR data as it is not considered appropriate when sample sizes are small (here three biological replicates for each line). Instead, in each figure we show the data for each of the three biological replicates as well as their average. Otherwise the exact N for each experimental group is indicated in the figure legend or displayed on each data table.

(For large datasets, or papers with a very large number of statistical tests, you may upload a single table file with tests, Ns, etc., with reference to sections in the manuscript.)

**Group allocation**

- Indicate how samples were allocated into experimental groups (in the case of clinical studies, please specify allocation to treatment method); if randomization was used, please also state if restricted randomization was applied
- Indicate if masking was used during group allocation, data collection and/or data analysis

Please outline where this information can be found within the submission (e.g., sections or figure legends), or explain why this information doesn’t apply to your submission:

Not relevant to this study, experimental groups consist of plants of different genotypes.

**Additional data files (“source data”)**

- We encourage you to upload relevant additional data files, such as numerical data that are represented as a graph in a figure, or as a summary table
- Where provided, these should be in the most useful format, and they can be uploaded as “Source data” files linked to a main figure or table
- Include model definition files including the full list of parameters used
- Include code used for data analysis (e.g., R, MatLab)
- Avoid stating that data files are “available upon request”

Please indicate the figures or tables for which source data files have been provided:

Not relevant to this study.
